# 2,4-dinitrophenol intoxication: Lack of adsorption despite prior literature suggesting efficacy

**DOI:** 10.1016/j.toxrep.2026.102350

**Published:** 2026-09-30

**Authors:** Florian A. Kammerstetter, Mattia M. Müller, Jens Martens-Lobenhoffer, Stefanie M. Bode-Böger, Jan T. Kielstein, Sascha David, Alix Buhlmann

**Affiliations:** a Institute of Intensive Care Medicine, University Hospital Zurich & University of Zurich, Zurich, Switzerland; b Institute of Clinical Pharmacology, Otto-von-Guericke University, Magdeburg, Germany; c Institute of Pharmacology, Toxicology and Clinical Pharmacy (IPTK), Technical University Braunschweig, Braunschweig, Germany; d Nephrology and Hypertension, Medical School Hannover, Hannover, Germany

**Keywords:** DNP, Clearance, Hemoadsorption, Intoxication, Removal Saturation

## Abstract

2,4-Dinitrophenol (DNP) is a potent mitochondrial uncoupler associated with life-threatening hypermetabolism and refractory hyperthermia. It remains widely available online despite repeated regulatory warnings and numerous reported fatalities. We report a severe DNP intoxication with multi-organ dysfunction in a 22-year-old man. Continuous veno-venous hemodialysis combined with CytoSorb® hemoadsorption (HA) was initiated as previous reports suggest a benefit associated with these techniques. Serial measurements were performed to assess extracorporeal clearance, which was calculated using pre- and post-adsorber blood sampling. No meaningful reduction in plasma concentration or adsorber-based clearance was observed, indicating minimal extracorporeal elimination of DNP. The patient improved under supportive care including active cooling and recovered fully. This case highlights that declining serum concentration alone are not sufficient to evaluate effectiveness of HA for toxin removal and emphasize the importance to use EXTRIP dialyzability criteria. Overall, HA with CytoSorb® demonstrated no measurable DNP elimination.

## Highlights

• intoxication can be lethal by uncoupling mitochondrial oxidative phosphorylation.
• it has a narrow therapeutic index and no antidote.
• it is not detected on routine toxicology screening.
• removal by HA is mainly hampered by a concentration dependent protein binding.
• Plasma concentration alone is misleading to evaluate adsorption.

## Graphical Abstract

**Figure:**
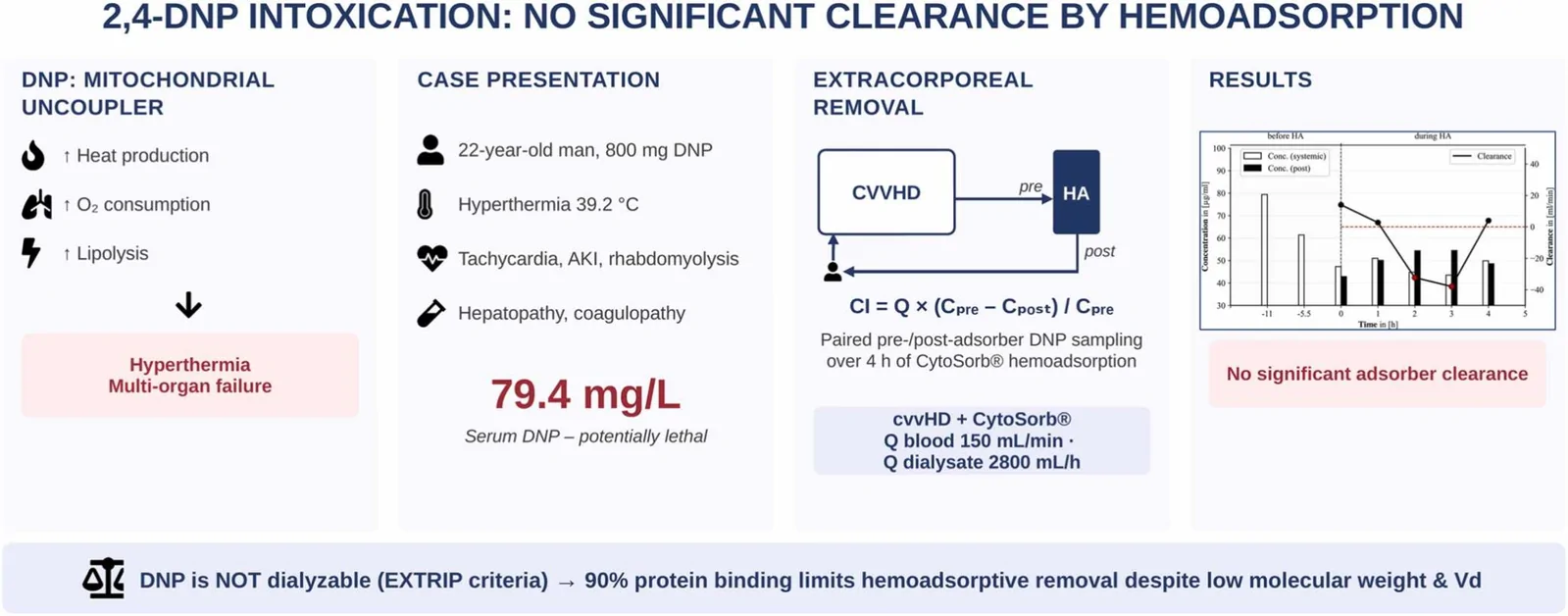

## 1 Introduction

2,4-Dinitrophenol (DNP) is a highly toxic mitochondrial uncoupler that disrupts oxidative phosphorylation, leading to severe hypermetabolism, refractory hyperthermia, and multi-organ failure, classically described as a state in which patients effectively “burn calories to death” [1]. Despite its narrow therapeutic index and numerous associated fatalities, DNP remains widely available online and reports of fatal intoxications have recently increased again. An intoxication may not only occur after acute overdose but also following cumulative exposure during prolonged use or dose escalation of the often sold weight-loss supplement [1]. The diagnosis is challenging, as DNP is not detected in routine toxicology screening, making clinical suspicion crucial - particularly in cases of unexplained hyperthermia and high anion gap metabolic acidosis. To date no specific antidote exists, and current management is exclusively supportive, with early active cooling being a cornerstone, as antipyretics are ineffective [1]. DNP has a low molecular weight (184 g/mol) and a small volume of distribution (0.3 L/kg) [2,3] but is highly protein-bound (>90%), although this is concentration-dependent. This seems to be a major limiting factor to extracorporeal elimination despite some publications suggesting a therapeutic benefit, based solely on repeated serum concentration [4, 5, 6]. Classifying dialyzability on serial serum measurements is notoriously misleading as they do not account for volume of distribution, endogenous elimination, and extracorporeal elimination as stated by the EXTRIP workgroup. While treating a patient with potentially lethal DNP concentration, we assessed CytoSorb®’s efficacy using repeated clearance measurements and found no significant toxin adsorber clearance. To our knowledge, this is the first report providing quantitative data on CytoSorb®’s DNP clearance.

## 2 Case Presentation

A 22-year-old man (BMI of 25.1 kg/m^2^, weight 79 kg, height 184 cm) was referred to our emergency department (ED) with reduced consciousness, hyperthermia and tachypnoea. In the morning his mother had last seen him normal. Around 10 am the same day he had taken 800 mg of DNP with no other medication. Long-term medication consisted of brexpiprazol (daily 4 mg p.m.), bupropion retard (daily 300 mg p.m.) and illegal DNP, usually three doses of 300 mg. He used it for weight loss while attempting to increase muscle mass and purchased it online. Around 5:30 pm his mother found him in a markedly reduced general condition and at 8 pm she brought him to a regional hospital. Due to a rapid deterioration with progressive hyperthermia and arterial hypertension, he was urgently transferred to our tertiary care center. His medical history was notable for polysubstance abuse, including anabolic steroids and recreational drugs such as cocaine, MDMA, LSD, opioids, alcohol and cannabis. Except for depression, there was no relevant chronic medical illness.

On arrival at our ED, he presented with a Glasgow Coma Scale (GCS) score of 10 but agitated. He was tachypnoeic (30 breaths/min) and tachycardic (125 beats/min), severely hypertensive with systolic blood pressure values exceeding 200 mmHg, and hyperthermic with a core temperature of 39.2 °C despite ongoing cooling measures. Treatment at the regional hospital had included administration of approximately 6 L of cooled crystalloids (Ringerfundin® and glucose solutions). No other pathologies, markly no skin color changes were noted. Antihypertensive therapy with 15 mg urapidil and 150 µg clonidine was administered. Agitation was treated with 1 mg lorazepam and 1 mg midazolam. An additional 4 L of Ringerfundin® was infused and external cooling intensified using ice packs.

Laboratory investigations revealed multiorgan dysfunction with acute kidney injury (AKI) I, hepatopathy with coagulopathy, severe rhabdomyolysis and a respiratory compensated metabolic acidosis with lactate in the normal range (Table 1). Cerebral computed tomography showed no pathology. The patient was admitted to the intensive care unit (ICU) for further treatment. Subsequent laboratory investigations revealed a potentially lethal total DNP serum concentration of 79.4 mg/l. Otherwise urine toxicology was only positive for benzodiazepine, which he received in the ED before urine sampling (negative: amphetamine, barbiturate, cannabis, cocaine, LSD, methaqualone, methadone, opioids, phencyclidine).

**Table 1:** patients laboratory findings at hospital admission.

Parameter	Result	Normal range	Unit
Albumin	38	40 – 49	g/l
Protein	57	60 – 80	g/L
AST	2070	< 50	U/l
ALT	186	< 50	U/l
INR	1.6	< 1.2	
Factor V	54	50 – 150	%
Potassium	4.4	3.4 – 4.5	mmol/l
Creatinine kinase	6765	< 190	U/l
Myoglobin	25,865	28 – 72	U/l
Hemoglobin	130	134 – 170	g/l
Hematocrit	0.387	0.4 – 0.5	l/l
pH	7.428	7.35 – 7.45	
pCO_2_	2.1	4.66 – 6.38	kPa
Lactate	1.1	0.4 – 0.8	mmol/l
HCO_3_	15.3	22.2 – 28.3	mmol/l
BE	-13.5	-3.2 – 1.8	mmol/l
Blood alcohol	< 2.2	< 2.2	mmol/l

Upon ICU admission, the patient remained somnolent but intermittently agitated with a GCS of 10. He had a temperature of 38.3°C, was tachycardic (133 beats/min), tachypnoeic (33 breaths/min), and normotensive (117/63 mmHg) under i.v. anti-hypertensive therapy. Transthoracic echocardiography demonstrated a hyperdynamic left ventricle with preserved systolic function and no structural abnormalities. On the second day, AST (2038 U/l) and creatine kinase concentration (93,483 U/l) peaked and myoglobin had already peaked at admission. Due to literature suggesting extracorporeal removal, we used a CytoSorb® adsorber connected in series with a continuous veno-venous hemodialysis (CVVHD), indicated due to rhabdomyolysis and AKI. To calculate DNP clearance pre- and post-adsorber DNP concentration were measured (CI = Q x (1 - Hct) x (C_pre_ – C_post_) / C_pre_; Q= blood flow; Hct =Hematocrit C_pre_= pre-adsorber DNP concentration; C_post_= post-adsorber DNP concentration) (Fig. 1). We used a Fresenius Multifiltrate Pro Ci-Ca dialyzer with the CytoSorb® installed downstream, a dialysate flow of 2800 ml/h and blood flow of 150 ml/min. Moreover, the patient received supportive therapy, including volume resuscitation and for the first 60 h he needed an external cooling device (ArcticSun™) to maintain body temperature bellow 38°C. The patient improved steadily until transfer to the ward on the fourth day, fully awake, calm, oriented, and cooperative with a fully recovered kidney function.

**Fig. 1:**
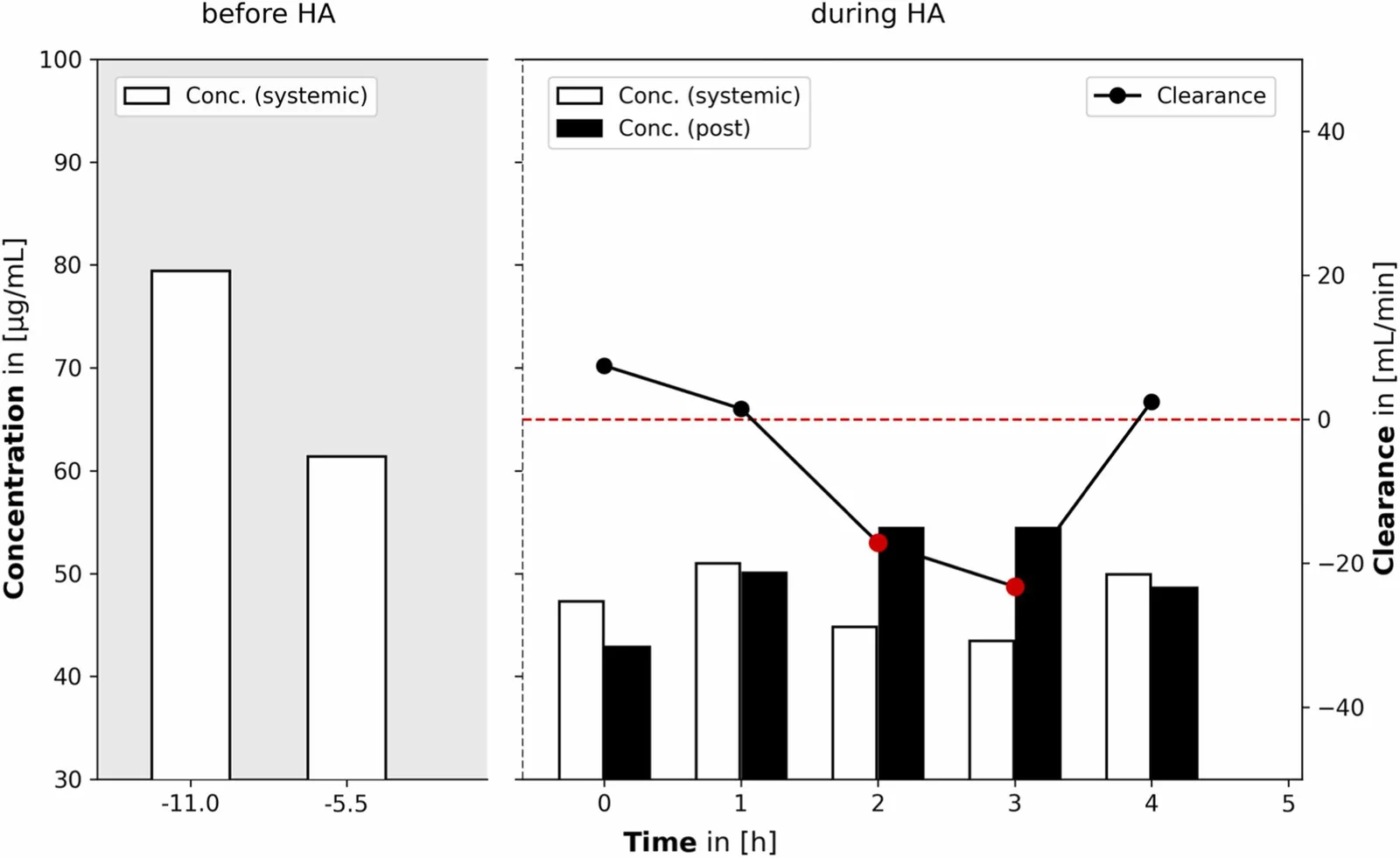
course of total DNP serum concentration before (left - natural course, no indication for dilution hematocrit (41.7–40.5%) and albumin (38–34 g/l) mostly stable from before HA start) and during 4 h of HA, as well as CytoSorb®’s DNP clearance.

## 3 Discussion

DNP intoxication was first described in 1919 among French workers involved in amunition manufacturing during the World War I [1,7]. It predominantly acts as a proton ionophore and uncouples mitochondrial oxidative phosphorylation by dissipating the proton gradient through the inner mitochondrial membrane without producing adenosine triphosphate (ATP), releasing excess heat. Basal metabolism, especially of lipids and oxygen consumption are markedly increased [1]. The clinical picture of acute DNP intoxication is characterized by rapidly evolving hyperthermia, a pronounced sympathomimetic-like syndrome and biochemical features of global cellular energy failure. Most frequently described symptoms are hyperthermia, nausea, vomiting, confusion, dizziness, headache, agitation, breathing difficulties, back and abdominal pain, tachycardia, diaphoresis, and tachypnoea, as well as yellow skin alterations and rashes [1,8]. Serum concentration of ≥ 28 µg/ml have been described as lethal [9].

There is no specific antidote, and management relies on early recognition and rapid administration of supportive care [1]. Because of some physicochemical properties of DNP, as it is hydrophobic and has a molecular mass of just 184.11 g/mol (= 0.18411 kDa) [2,3], extracorporeal toxin removal by HA has been described as a potential treatment option. Moreover, the low volume of distribution of 0,3 kg/L seems to support this notion, however concentration dependent protein binding exceeding 90% at toxic concentration cast doubt on the usefulness of extracorporeal therapies in DNP intoxication. Animal data indicate that albumin binding of DNP remains above 90% even at highly toxic blood levels, suggesting that the nonlinearity in binding, while measurable, is insufficient to substantially increase the unbound fraction in the way observed with valproic acid at supratherapeutic concentrations. Previous publications evaluating HA in DNP intoxication have described decreasing plasma DNP concentrations over time as well as clinical improvement during treatment, particularly regarding hyperthermia and sympathomimetic symptoms [4, 5, 6]. However, these observations were primarily based on serial serum measurements and clinical course rather than quantitative extracorporeal clearance assessments. To demonstrate clinically relevant extracorporeal toxin removal, the EXTRIP criteria for dialyzability should be fulfilled, requiring either > 30% substance removal or an extracorporeal-to-total-clearance ratio exceeding 75%, as a measurable extracorporeal clearance must represent a substantial proportion of total body clearance relative to endogenous elimination [10]. Declining plasma concentrations alone do not necessarily demonstrate effective extracorporeal toxin removal, as similarly observed for substances with high volumes of distribution such as tricyclic antidepressants. Thus, current evidence remains insufficient to conclude that extracorporeal therapies substantially enhance DNP elimination. Therefore, in our patient’s case, to assess the efficacy of DNP removal, we calculated DNP clearance. There was neither a significant decrease in DNP plasma concentration during HA nor significant toxin adsorber clearance comparing simultaneously drawn pre- and post-adsorber blood samples for DNP measurement.

The CytoSorb® cartridge characteristics are by large comparable to the HA-350 adsorber used in the above mentioned reports. It consists of cross-linked divinylbenzene and polyvinylpyrrolidone copolymers and adsorbs hydrophobic molecules below 55 kDa. The HA-330 adsorber consists of polystyrene divinylbenzene copolymer resins, adsorbing hydrophobic molecules in the 10–60 kDa range.

Our investigation demonstrated a non-dialyzability of DNP using Continuous veno-venous hemodialysis combined with CytoSorb® hemoadsorption according to the EXTRIP-criteria. Minor extraction below assay precision limits cannot be excluded, nor supportive effects of HA unrelated to toxin removal. Our findings challenge the frequently cited notion that CytoSorb® effectively removes protein-bound substances, suggesting instead that extensive protein binding limits adsorptive clearance. Overall, this report highlights the clinical relevance of pharmacokinetics and toxicokinetics. For DNP, low molecular weight and low volume of distribution combined with protein binding of > 90%, which does not significantly decrease at high concentration [3] make it a poor candidate for extracorporeal removal, with the potential exception of high volume plasma exchange.

## 4 Ethics approval and consent to participate

The patient had a signed informed consent for further use of health-related data.

## 5 Authors' contributions

AB and SD are responsible for the conception and design of the work; FK and AB acquired the data, JML and SBB measured DNP concentration. SD, FK and AB made substantial contributions to the interpretation of data. FK, SD and AB have drafted the work and all authors, including JK, JML, SBB, MM, substantially revised it.

All authors have approved the submitted version and agreed both to be personally accountable for the author's own contributions and to ensure that questions related to the accuracy or integrity of any part of the work, even ones in which the author was not personally involved, are appropriately investigated, resolved, and the resolution documented in the literature.

Authors: Florian A. Kammerstetter (FK), Jens Martens-Lobenhoffer (JML), Stefanie M. Bode-Böger (SBB), Jan T. Kielstein (JK), Mattia M. Müller (MM), Sascha David (SD), Alix Buhlmann (AB),

## Author statement

Sascha David is supported by an unrestricted research grant from Cytosorbents for a RCT to test CytoSorb in CRS (CYTORELEASE-Ex)

## CRediT authorship contribution statement

**Florian A. Kammerstetter:** Writing – original draft, Visualization, Data curation. **Mattia M. Müller:** Writing – review & editing, Methodology. **Jens Martens-Lobenhoffer:** Writing – review & editing, Methodology. **Stefanie M. Bode-Böger:** Writing – review & editing. **Jan T. Kielstein:** Writing – review & editing, Methodology. **Sascha David:** Writing – review & editing, Supervision, Resources, Conceptualization. **Alix Buhlmann:** Writing – review & editing, Supervision, Formal analysis, Data curation, Conceptualization.

## Consent for publication

The Patient gave consent for publication

## Guarantor

Alix Buhlmann takes responsibility for the content of the manuscript, including the data and analysis.

## Role of sponsor

Not applicable

## Financial / nonfinancial disclosures

None

## Declaration of Competing Interest

The authors declare the following financial interests/personal relationships which may be considered as potential competing interests:Sascha David reports a relationship with Cytosorbents that includes: funding grants. If there are other authors, they declare that they have no known competing financial interests or personal relationships that could have appeared to influence the work reported in this paper.

## Data Availability

Data will be made available on request.
